# Standardizing therapeutic parameters of acupuncture for pain suppression in rats: preliminary study

**DOI:** 10.1186/1472-6882-14-25

**Published:** 2014-01-15

**Authors:** Sujung Yeo, Hyungtaeck Lim, Ilwhan Choe, Sung-Hoon Kim, Sabina Lim

**Affiliations:** 1 Department of Acupuncture and Meridian, Graduate School of Applied Korean Medicine, Kyung Hee University, Seoul, Republic of Korea; 2 Research Group of Pain and Neuroscience, WHO Collaborating Centre, East–west Medical Research Institute, Kyung Hee University, Seoul 130-701, Republic of Korea

**Keywords:** Acupuncture, Therapeutic parameters, Pain suppression, Electrical nerve stimulation method (TENS), Diffuse noxious inhibitory controls (DNIC)

## Abstract

**Background:**

Despite acupuncture’s wide and successful use, it is still considered as lacking scientifically rigorous evidence, especially with respect to its effectiveness. To address this problem, it is necessary to re-examine the practice of acupuncture using scientific methodology. The standardization of acupuncture practices may offer a solution. As a preliminary step towards the standardization of acupuncture stimulation in animal experiments, this study attempted to clarify the various therapeutic parameters that contribute to acupuncture’s efficacy.

**Methods:**

This study identified specific acupoints, temporal point of needling, rotation of the needle, duration of acupuncture, and diameter of the needle as the parameters, through formalin test. In this test, acupuncture was performed on either the ST36 or LR2 point immediately after pain induction and 5 minutes after pain induction.

**Results:**

The formalin test yielded no significant suppression of pain in the case of ST36 and LR2 acupuncture stimulation immediately following pain induction. When acupuncture was applied 5 minutes after pain induction, however, the ST36 stimulation resulted in a significant decrease in pain, while the LR2 stimulation produced no change. The duration of acupuncture, but not the diameter of the needle, was also significant. As for the rotation of the needle, there was no significant difference in the pain reduction achieved in the rotation and non-rotation groups.

**Conclusions:**

We determined that specific acupoint, temporal point of needling, and duration of treatment are important factors in the inhibition of pain. These finding strongly suggest that in animal experiments, the application of a set of appropriate therapeutic parameters can significantly influence the outcome.

## Background

Acupuncture has been successfully used for clinical treatment in East Asian countries for numerous centuries. The effectiveness of the treatment, however, is still unclear and many challenges remain in proving its medicinal effect with scientific rigor. Since its introduction to the Western world in the early 1970s, the effect of acupuncture has been discussed, whether favorably or critically, along with newly published neuro-biological theories. Melzack et al. [1] presented the theory of gate control, which was an early model of acupuncture-induced pain suppression [1,2] and was eventually developed into the Transcutaneous Electrical Nerve Stimulation method (TENS) [3]. Le Bars discussed the idea of Diffuse Noxious Inhibitory Controls (DNIC) [4]. Bing argued that there was an affinity between the acupuncture-induced pain suppression effect and DNIC; yet the same authors deny the singular quality of acupuncture, implying that acupuncture stimulation is nothing but a kind of noxious inhibitory control [5,6]. Similarly, Cho et al. retracted their publication that had tried to prove the unique quality of acupoints through the use of fMRI but deny doing so [7].

When an acupoint is stimulated, two kinds of effects are expected: the effects due to the unique quality of the acupoint and the secondary effects, such as placebo and stress. Lao argues that the secondary effects alone do not account for the overall effect of acupuncture [8]. He insists that in order to explore the significant effects of acupuncture, it is necessary to identify the key therapeutic parameters that optimize the acupoints’ unique qualities [8]. Ceccherelli et al. [9] suggest several key therapeutic parameters; specific acupoints, number of needles, diameter of the needle, delivery method, depth of insertion, number of acupuncture sessions, and length of the clinical trial [9].

The studies reviewed so far, however, have been conducted with electrical acupuncture rather than manual acupuncture. Thus, Carlsson [10] suggests a need to look into the effects and mechanisms of manual acupuncture [10]. Moreover, in the studies on the unique qualities of acupoints, the question of how to eliminate secondary effects like placebo and stress still remains unanswered. This study investigated the therapeutic parameters of manual acupuncture using a patho-physiological animal model which can minimize the interference of non-specific effects. Animals are likely subject to less of a placebo effect than people. Patho-physiological animal models are necessary to observe the effects of treatment, which are not clearly displayed in non-patho-physiological models. Based on these facts, this study used a formalin test and manipulated specific acupoints, temporal point of needling, rotation of the needle, duration of acupuncture, and diameter of the needle as individual therapeutic parameters. Changes in the animals’ behavior (such as biting and licking the formalin injected area or shaking and jerking the formalin injected foot) were then observed to measure their pain response.

This study aimed to determine how individual therapeutic parameters affect the overall clinical effect of acupuncture. The results may highlight the importance of individual therapeutic parameters in acupuncture and strengthen the scientific rigor of acupuncture-related research.

## Methods

### Animal

The protocol for the animal experiment was reviewed and approved by the Animal Care and Use Committee of Kyung Hee University. Male Sprague–Dawley rats were used (Orient, Korea). Rats weighed 220–250 g. Rats were housed at room temperature (22-24°C) in standard 12 h light/dark cycles and given unlimited access to food and water. Throughout the experimental procedure, the animal care guidelines of the NIH were strictly observed.

### Needles

Stainless Steel needles (Hanglimseowon Medical Instruments, Korea; dia. 0.17 and 0.4 mm) were used. The inserted portion of the needle’s body (10 mm) was marked to render its length consistent (Additional file 1: Figure S1).

### Acupoints and acupuncture method

ST36 and LR2 acupoints were selected for acupuncture stimulation. ST36 has been used to alleviate pain in both animal experiments [11,12] and clinical trials [13]. LR2 is used to prevent increases in body temperature [14]. To familiarize rats with the treatment, each group was conditioned through the same handling as the experimental treatment for 5 days before the experiment. Rats were held by a human hand and received 10 min of acupuncture treatment, depending on their assigned group, every day starting 5 days before the beginning of the experiment. ST36 was taken 5 mm outside to the tibialis anterior (tibialis anterior muscle) at the line of distal beginning of tibial tuberosity. For LR2, the chosen point was on the dorsum of the hind foot between the first and second toes, proximal to the web margin. Acupuncture was performed on the side opposite (left) to the pain-induced side. The needle was inserted vertically 5.0 mm and 2.0 mm deep on ST36 and LR2, respectively.

### Formalin test

(1) Pain induction

 50 μl 0.9% saline + 2% formalin solution (Sigma, USA) was injected into the top of the right hind foot with a 30 gauge Hamilton syringe.

(2) Schemes of the test

**Figure 1:**
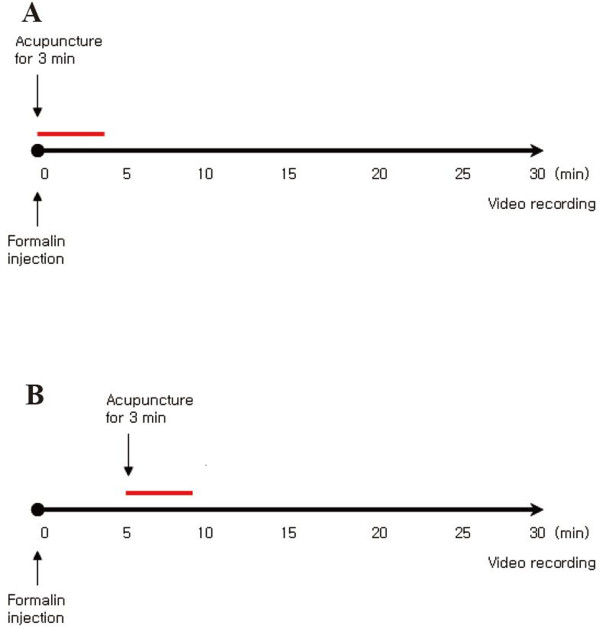
**Schemes of formalin test.** Scheme **(A)**: acupuncture was treated from the beginning of pain induction for three minutes. **(B)**: acupuncture was treated five minutes after pain induction. Behavior was recorded for thirty minutes.

 To observe the changes in the pain responses ensuing from the different kinds of acupoints and treatment starting times, rats were divided into four groups: ST36 + immediately-after-pain-induction, ST36 + 5-min-after-pain-induction, LR2 + immediately-after-pain-induction, and LR2 + 5-min-after-pain-induction. Each group was subjected to 3 min of acupuncture with a 0.17 mm diameter needle rotated 2 times per second for the first and the last minute (Figure 1).

 To examine the effect of the duration of acupuncture, rats were divided into three groups: Duration 0.5 min (30 seconds) (Duration 0.5), Duration 1 min (Duration 1), and Duration 3 min (Duration). The Duration 3 group received rotation treatment 2 times per second for the first and the last minute. Rotation was performed in the Duration 0.5 and Duration 1 groups for the entire duration of treatment, and a 0.17 mm diameter needle was used for all groups. To evaluate the effect of the needle’s diameter, rats were divided into Diameter 0.17 mm and Diameter 0.40 mm groups for acupuncture treatment of 3 minutes with rotation as described for the Duration 3 group (Table 1).

 To observe the effect of needle rotation, the rotation group received needle rotations 2 times per second for the first and last 1 min of a 3 min treatment with a 0.l7 mm diameter needle. The non-rotation groups did not receive any rotation for the duration of the trial.

(3) Pain response analysis

 Behavioral indexes, such as biting and licking the formalin injected area or shaking and jerking the formalin injected foot, were interpreted as pain responses. These behavioral indexes were collected by recording rats on video and then analyzed with SMISyncW program (Seoul, Korea). The time a particular pain response started (Ts) and the time it ended (Te) were manually measured. The difference (Tr = Te – Ts) was calculated as the total response time. After the injection the rats’ behaviors were observed for 30 min, not including the time elapsed during the actual insertion of the needle. As for the control group, rats were lightly grabbed by a human hand for the same period as the actual application of acupuncture in order to give the same stimulation that the other groups received.

**Table 1:** Groups of formalin test

**Parameter**	**Groups**		
Acupoint and temporal point of needling	Right after formalin injection		ST36
			LR2
	5 min after formalin injection		ST36
			LR2
Rotating of needle	Rotation		Non-rotation
Duration of needling	Duration 0.5	Duration 1	Duration 3
Diameter of needle	0.17 mm		0.40 mm

Control group of each parameter was not included in this table.

### Statistical data analysis

All data were presented as mean ± SE. Statistical analyses of the data were conducted with a one way Analysis Of Variance (ANOVA) and a t-Test using SigmaStat for Windows version 3.10. The level of significance was less than 0.05. Fisherman LSD was used for a post-hoc of one way ANOVA.

## Results

### Basic pain response in formalin test

**Figure 2:**
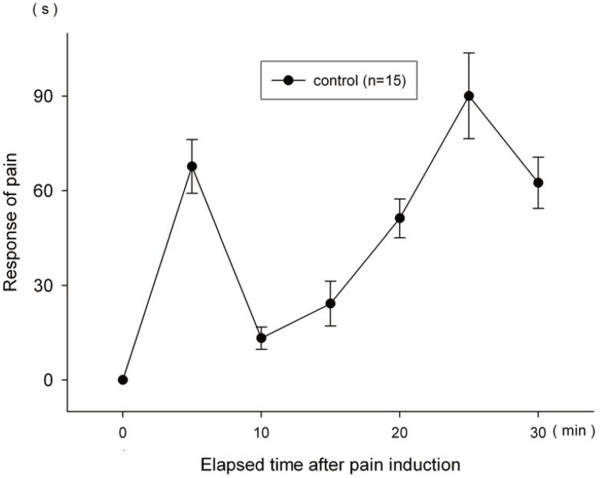
**Pain responses in the formalin test.** Pain response of phase 1 increases from start till 5 minutes and decreases till 10 minutes. In case of phase 2, response increases gradually till 25 minutes and starts to decrease thereafter.

The data accumulated in 5-minute increments were organized up to 10 minutes from the 0 minute point for the first phase and up to 20 minutes from 10 minutes for the second phase. Pain response in phase 1 increased from the start untill 5 minutes and then decreases untill 10 minutes. In phase 2, responses increased gradually untill 25 minutes and started to decrease thereafter (Figure 2).

### Pain responses according to acupoint and temporal point of needling

(1) Pain responses when acupuncture began immediately after pain induction.

**Figure 3:**
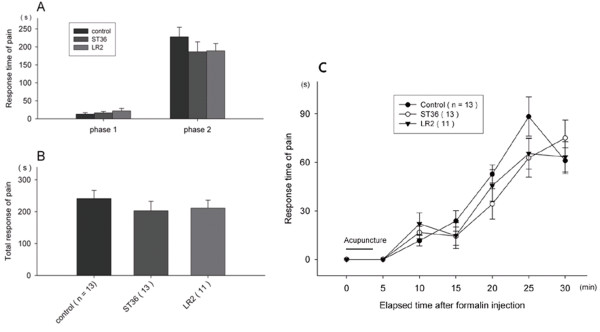
**Pain responses in the formalin test when acupuncture began immediately after pain induction.** Pain responses of phase 1 and phase 2 were presented in **(A)** and total pain response was presented in **(B)**. Sequential pain response was presented in **(C)**. The black bar labeled "Acupuncture" indicates the 5 minute time interval during which the 3 minute treatment occurred for all mice. All data are presented as mean ± SE. No significant difference was found in pain responses compared to the control group.

 The formalin test yielded no significant suppression of pain in the case of the ST36 or LR2 acupuncture stimulation that immediately followed the injection of formalin (Figure 3). In this group, the typical 10-minute sudden increase and subsequent fall which were appeared in formalin test group was not observed.

(2) Pain responses when acupuncture began after 5 min after pain induction.

**Figure 4:**
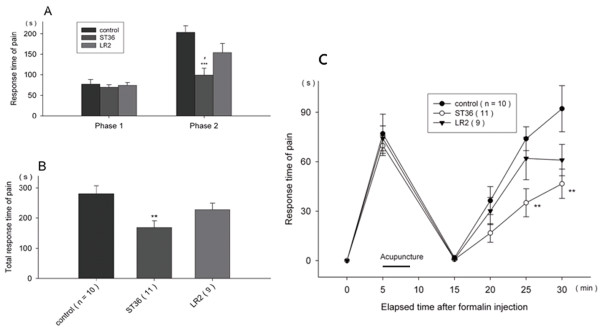
**Pain responses in the formalin test when acupuncture began 5 min after pain induction.** Responses according to phase were presented in **(A)** and total response was presented in **(B)**. Sequential pain response was presented in **(C)**. All data are presented as mean ± SE. The black bar labeled "Acupuncture" indicates the 5 minute time interval during which the 3 minute treatment occurred for all mice. **p < 0.01 and ***p < 0.001 when compared to control group, #p < 0.05 when compared to LR2 group.

 In this group, the typical 10-minute sudden increase and subsequent fall which appeared in the formalin test group were observed. When acupuncture was applied 5 minutes after pain induction, the ST36 stimulation resulted in a significant pain decrease while the LR2 stimulation produced no decrease (Figure 4).

### Pain responses in the formalin test related to duration of treatment

**Figure 5:**
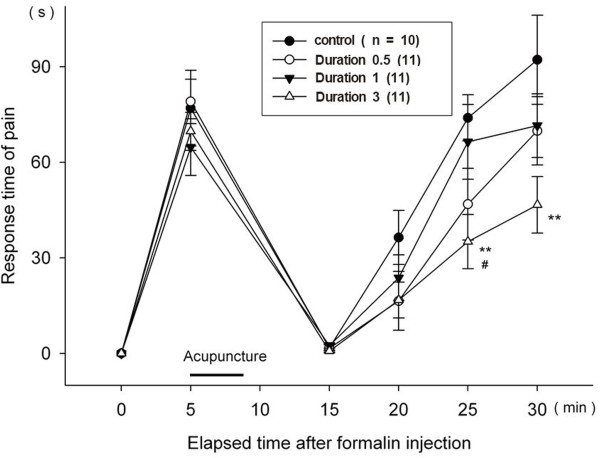
**Sequential pain responses in the formalin test related to duration of treatment.** The treatment occurred 5 minutes after pain induction. In all of the following figures, the response could not be measured from 5 minute to 10 minute. The black bar labeled "acupuncture" indicates the 5 minute time interval during which the 30 second, 1 minute, or 3 minute treatment occurred for all mice. All data are presented as mean ± SE. **p < 0.01 when compared to the control group. #p < 0.05 when compared to the Duration 1 group.

The needle was inserted 5 minutes after pain induction. The different durations of acupuncture also produced different pain relief results. The duration 3 group (for which the needle was left inserted for 3 minutes on the acupoint) showed a significant decrease in pain from 20 min until 30 min, while the Duration 0.5 and Duration 1 groups failed to show any pain decrease (Figure 5).

### Pain responses related to the diameter of the needle

**Figure 6:**
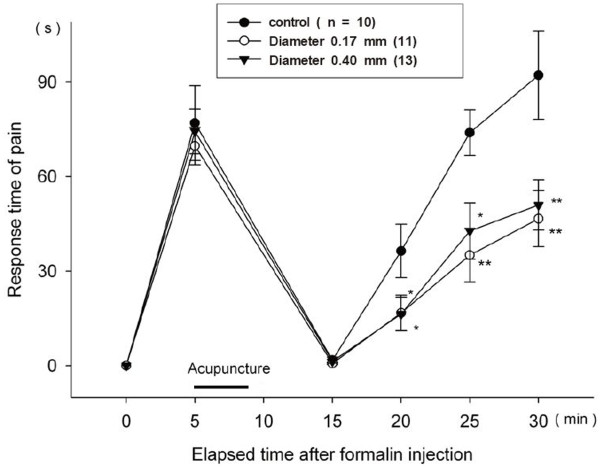
**Sequential pain responses in the formalin test related to needle diameter.** The needle was inserted 5 minutes after pain induction. The black bar labeled "Acupuncture" indicates the 5 minute time interval during which the 3 minute treatment occurred for all mice. All data are presented as mean ± SE. *p < 0.05 and **p < 0.01 when compared to the control group.

The needle was inserted 5 minutes after pain induction. Both the 0.17 m and 0.40 m groups showed significant pain decreases, however, the diameter of the needle did not make a substantial difference in relieving pain (Figure 6).

### Pain responses related to needle rotation

**Figure 7:**
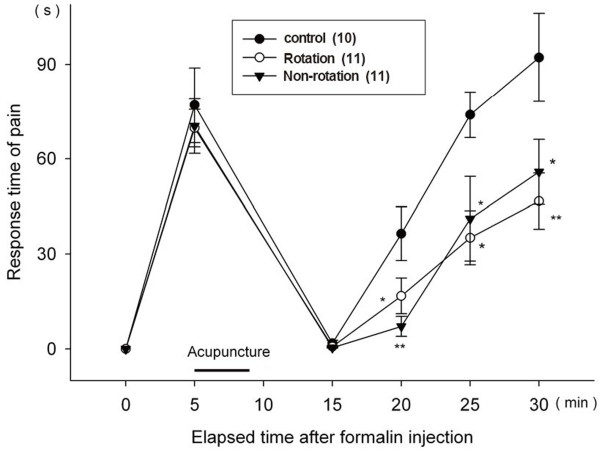
**Sequential pain responses in the formalin test related to needle rotation.** The black bar labeled "acupuncture" indicates the 5 minute time interval during which the 3 minute treatment occurred for all mice. The needle was inserted 5 minutes after pain induction. In the rotation group, the needle was rotated two times per second in the first minute after the start of pain induction and for another two times per second in the last minute of acupuncture stimulation. The non-rotation group received acupuncture for 3 minutes without needle rotation. All data are presented as mean ± SE. *p < 0.05 and **p < 0.01 when compared to the control group.

The needle was inserted 5 minutes after pain induction. In the rotation group, the needle was rotated two times per second in the first minute after the start of pain induction and for another two times per second in the last minute of acupuncture stimulation. The non-rotation group received acupuncture for 3 minutes without needle rotation. Both groups showed similar decreases in pain (Figure 7). Thus needle rotation did not have a substantial role in relieving pain.

## Discussion

This preliminary study aimed to determine how individual therapeutic parameters affect the overall clinical effect of acupuncture. The results may highlight the importance of individual therapeutic parameters in acupuncture and strengthen the scientific rigor of acupuncture-related research. In general, acupuncture is applied to relieve patients’ acute pain. This study, therefore, was designed to measure the different effects of acupuncture treatment delivered when the amount of pain reaches its climax versus when the pain has not yet reached its climax.

### Basic pain response in formalin test

A formalin test is used to measure for 30 minutes or up to 1 hour the pain response that is divided into the early phase and the late phase, or Phase 1 and Phase 2. In this study the data were accumulated every 5 min. The accumulated response at 5 min was about 68 sec response time. From 5 to 10 min, it decreased to 13 seconds, and after 10 min it gradually increased. From 20 to 25 min it reached the peak of 90 seconds and then decreased thereafter.

### Pain responses according to acupoint and temporal point of needling

The results show that in the groups immediately receiving treatment neither ST36 nor LR2 showed a significant effect in pain suppression; in the groups receiving treatment after 5 min after the formalin injection, however, ST36 demonstrated a significant pain suppression effect. Yet, even in the 5-min-after-pain-induction, LR2 showed no significant effect. In previous research, ST36 has been used to alleviate pain in both animal experiments [11,12] and clinical trials [13]. The current results further support the notion that ST36 could alleviate pain. It has been suggested that this effect is mediated by activation of the opioidergic and serotonergic systems [11]. On the other hand, LR2 was used to deter increases in body temperature [14]. These results demonstrated that unique qualities of individual acupoints exist. The results also highlight the importance of the starting time of acupuncture intervention. 5 min after the formalin injection was the peak time of first phase pain response in the formalin test (Figure 2). In the 5-min-after-pain-induction group, ST36 demonstrated a significant pain suppression effect, but not in the immediately-after-pain-induction group. This suggests that acupuncture stimulation at the peak time of acute pain may be more effective.

### Pain responses in the formalin test related to duration of treatment

Unlike the Duration 0.5 and Duration 1 groups, only the Duration 3 group yielded a significant effect. This result suggests that duration is an important therapeutic parameter of acupuncture. To allow an adequate amount of time to observe the rats’ behavior in response to the formalin injections, the longest duration was limited to 3 min. In human research, it has been found that acupuncture stimulation for 40 min achieves the strongest and longest lasting effects [15]. The duration of 3 min, therefore, cannot be interpreted as the optimal duration. To discover the optimal duration, it may be necessary to develop a new formalin test with a longer duration of acupuncture stimulation. It should be pointed out that continuous acupuncture stimulation for more than 1–2 h may lead to a diminution of analgesic effect, a phenomenon known as "acupuncture tolerance". One of the underlying mechanisms is the accelerated release of central cholecystokinin octapeptide which acts against the analgesic effect of the endogenous opioid peptides [16,17]. Therefore, excessive duration of acupuncture stimulation is not recommended [18].

### Pain responses related to the diameter of the needle

A significant pain suppression effect was observed in both the diameter 0.17 mm and diameter 0.40 mm groups. This result suggests that it is necessary to further study the effect of needle diameters with a greater variety of diameters in order to prove that needle thickness is a genuine therapeutic parameter. Such research is especially necessary considering the modern trend of using thinner needles to lessen the pain inflicted by inserting the needle. Before technology helped make acupuncture needles slimmer, the needles were much thicker than their modern versions. It has not yet been determined whether the same effect described in ancient discourses on acupuncture could be obtained with the thinner modern needles.

### Pain responses related to needle rotation

The rotation and non-rotation groups showed similar pain suppression effects. This result partly contradicts some of the recent research papers that report significant differences between the effects of application and non-application of rotation [19,20]. Previous research found that needle rotation increases pull-out force through connective tissue winding, which modifies the biomechanical behavior of soft tissue [21]. A preliminary study reported that introduction of needle rotation significantly increased deep, dull, heavy sensations related to *deqi*[22]. *Deqi* is suggested to be the main mechanism producing effects of acupuncture [23,24]. In our study, acupuncture stimulation duration was only 3 min, which could be too brief to gather *deqi* induced by rotation. Therefore, further study is needed of longer acupuncture stimulation with and without needle rotation.

## Conclusion

This study was designed to assess how individual parameters affect the overall clinical effect of manual acupuncture. Through these results, we determined that the specific acupoints, temporal point of needling and duration of treatment are important deciding factors regarding the inhibitory effect of pain. These findings strongly suggest that in animal experiments, if adjusted accordingly, the application of a set of appropriate therapeutic parameters can significantly influence the outcome. Therefore, more extensive experiments are needed to optimized individual analgesic parameters.

## Competing interests

The authors declare that they have no competing interests to disclose.

## Authors’ contributions

SY and HL conceived and designed the study and carried out many of the culture experiments, analyzed and interpreted the data, and drafted the manuscript. IC performed some of the experiments and data analysis, and contributed to the drafting of the manuscript. SHK was involved in drafting and revising of the manuscript. SL was involved in the conception and design of the study and the supervision of experiments. All authors read the manuscript, contributed to its correction, and approved the final version.

## Pre-publication history

The pre-publication history for this paper can be accessed here:

http://www.biomedcentral.com/1472-6882/14/25/prepub

## Supplementary Material

Additional file 1: Figure S1 Marked needle’s body. The inserted portion of the needle’s body (10 mm) was marked to render its length consistent. Click here for file

## References

[B1] Melzack R Wall PD Pain mechanisms: a new theory Science 1965 150 3699 971 979 10.1126/science.150.3699.971 5320816

[B2] Melzack R Prolonged relief of pain by brief, intense transcutaneous somatic stimulation Pain 1975 1 4 357 373 10.1016/0304-3959(75)90073-1 141644

[B3] Melzack R Wall PD Acupuncture and transcutaneous electrical nerve stimulation Postgrad Med J 1984 60 710 893 896 10.1136/pgmj.60.710.893 6334851 PMC2418088

[B4] Le Bars D Dickenson AH Besson JM Diffuse noxious inhibitory controls (DNIC). I. Effects on dorsal horn convergent neurones in the rat Pain 1979 6 3 283 304 10.1016/0304-3959(79)90049-6 460935

[B5] Bing Z Villanueva L Le Bars D Acupuncture-evoked responses of subnucleus reticularis dorsalis neurons in the rat medulla Neuroscience 1991 44 3 693 703 10.1016/0306-4522(91)90088-6 1754056

[B6] Bing Z Villanueva L Le Bars D Acupuncture and diffuse noxious inhibitory controls: naloxone-reversible depression of activities of trigeminal convergent neurons Neuroscience 1990 37 3 809 818 10.1016/0306-4522(90)90110-P 2247225

[B7] Cho ZH Chung SC Jones JP Park JB Park HJ Lee HJ Wong EK Min BI New findings of the correlation between acupoints and corresponding brain cortices using functional MRI (Retraction of vol 95, pg 2670, 1998) Proc Natl Acad Sci USA 2006 103 27 10527 10527 9482945 10.1073/pnas.95.5.2670 PMC19456

[B8] Lao L Zhang RX Zhang G Wang X Berman BM Ren K A parametric study of electroacupuncture on persistent hyperalgesia and Fos protein expression in rats Brain Res 2004 1020 1–2 18 29 15312783 10.1016/j.brainres.2004.01.092

[B9] Ceccherelli F Gagliardi G Rossato M Giron G Variables of stimulation and placebo in acupuncture reflexotherapy J Altern Complement Med 2000 6 3 275 279 10.1089/acm.2000.6.275 10890338

[B10] Carlsson C Acupuncture mechanisms for clinically relevant long-term effects–reconsideration and a hypothesis Acupunct Med 2002 20 2–3 82 99 12216606 10.1136/aim.20.2-3.82

[B11] Erthal V da Silva MD Cidral-Filho FJ Santos AR Nohama P ST36 laser acupuncture reduces pain-related behavior in rats: involvement of the opioidergic and serotonergic systems Lasers Med Sci 2013 28 5 1345 1351 10.1007/s10103-012-1260-7 23291880

[B12] Kang SY Kim CY Roh DH Yoon SY Park JH Lee HJ Beitz AJ Lee JH Chemical stimulation of the ST36 acupoint reduces both formalin-induced nociceptive behaviors and spinal astrocyte activation via spinal alpha-2 adrenoceptors Brain Res Bull 2011 86 5–6 412 421 21889580 10.1016/j.brainresbull.2011.08.012

[B13] Melchart D Streng A Hoppe A Brinkhaus B Becker-Witt C Hammes M Irnich D Hummelsberger J Willich SN Linde K The acupuncture randomised trial (ART) for tension-type headache–details of the treatment Acupunct Med 2005 23 4 157 165 10.1136/aim.23.4.157 16430123

[B14] Son YS Park HJ Kwon OB Jung SC Shin HC Lim S Antipyretic effects of acupuncture on the lipopolysaccharide-induced fever and expression of interleukin-6 and interleukin-1 beta mRNAs in the hypothalamus of rats Neurosci Lett 2002 319 1 45 48 10.1016/S0304-3940(01)02538-1 11814650

[B15] Cheing GL Tsui AY Lo SK Hui-Chan CW Optimal stimulation duration of tens in the management of osteoarthritic knee pain J Rehabil Med 2003 35 2 62 68 10.1080/16501970306116 12691335

[B16] Han JS Ding XZ Fan SG Cholecystokinin octapeptide (CCK-8): antagonism to electroacupuncture analgesia and a possible role in electroacupuncture tolerance Pain 1986 27 1 101 115 10.1016/0304-3959(86)90227-7 3491355

[B17] Han JS Cholecystokinin octapeptide (CCK-8): a negative feedback control mechanism for opioid analgesia Prog Brain Res 1995 105 263 271 7568886 10.1016/s0079-6123(08)63303-8

[B18] Han JS Acupuncture analgesia: areas of consensus and controversy Pain 2011 152 3 S41 S48 10.1016/j.pain.2010.10.012 21078546

[B19] Kim SK Moon HJ Na HS Kim KJ Kim JH Park JH Lee SH Rhim SS Lee SG Min BI The analgesic effects of automatically controlled rotating acupuncture in rats: mediation by endogenous opioid system J Physiol Sci 2006 56 3 259 262 10.2170/physiolsci.SC002706 16839460

[B20] Kong J Fufa DT Gerber AJ Rosman IS Vangel MG Gracely RH Gollub RL Psychophysical outcomes from a randomized pilot study of manual, electro, and sham acupuncture treatment on experimentally induced thermal pain J Pain 2005 6 1 55 64 10.1016/j.jpain.2004.10.005 15629419

[B21] Langevin HM Churchill DL Wu J Badger GJ Yandow JA Fox JR Krag MH Evidence of connective tissue involvement in acupuncture FASEB J 2002 16 8 872 874 11967233 10.1096/fj.01-0925fje

[B22] Park JJ Akazawa M Ahn J Beckman-Harned S Lin FC Lee K Fine J Davis RT Langevin H Acupuncture sensation during ultrasound guided acupuncture needling Acupunct Med 2011 29 4 257 265 10.1136/aim.2010.003616 21642648 PMC4196666

[B23] Pariente J White P Frackowiak RS Lewith G Expectancy and belief modulate the neuronal substrates of pain treated by acupuncture Neuroimage 2005 25 4 1161 1167 10.1016/j.neuroimage.2005.01.016 15850733

[B24] Liu S Zhou W Ruan X Li R Lee T Weng X Hu J Yang G Activation of the hypothalamus characterizes the response to acupuncture stimulation in heroin addicts Neurosci Lett 2007 421 3 203 208 10.1016/j.neulet.2007.04.078 17574746

