# Supplementary figures and images for: Standardizing therapeutic parameters of acupuncture for pain suppression in rats: preliminary study
Source: BMC Complement Altern Med. 2014 Jan 15;14:25. doi: 10.1186/1472-6882-14-25 (PMC3897986; doi:10.1186/1472-6882-14-25)

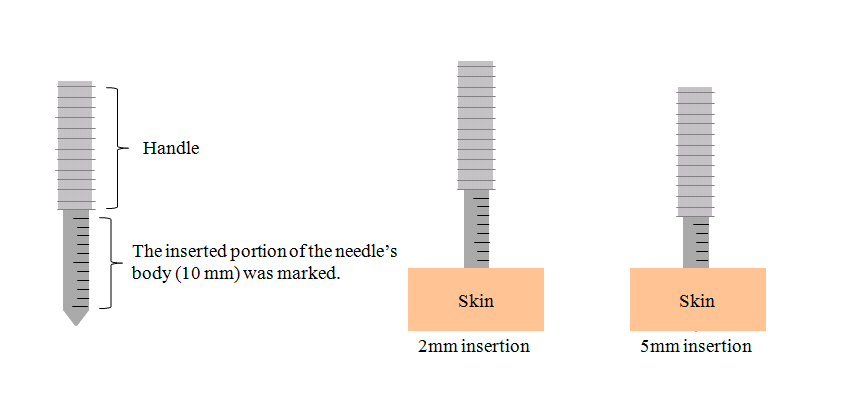

Supplement: Additional file 1: Figure S1 — Marked needle’s body. The inserted portion of the needle’s body (10 mm) was marked to render its length consistent. [file 1472-6882-14-25-S1.tiff]
